# Postgraduate education for Chinese medicine practitioners: a Hong Kong perspective

**DOI:** 10.1186/1472-6920-9-10

**Published:** 2009-02-19

**Authors:** Vincent CH Chung, Michelle PM Law, Samuel YS Wong, Stewart W Mercer, Sian M Griffiths

**Affiliations:** 1 School of Public Health, Chinese University of Hong Kong, HongKong SAR, PR China; 2 Section of General Practice & Primary Care, Division of Community-Based Sciences, Faculty of Medicine, University of Glasgow, Glasgow, UK

## Abstract

**Background:**

Despite Hong Kong government's official commitment to the development of traditional Chinese medicine (TCM) over the last ten years, there appears to have been limited progress in public sector initiated career development and postgraduate training (PGT) for public university trained TCM practitioners. Instead, the private TCM sector is expected to play a major role in nurturing the next generation of TCM practitioners. In the present study we evaluated TCM graduates' perspectives on their career prospects and their views regarding PGT.

**Method:**

Three focus group discussions with 19 local TCM graduates who had worked full time in a clinical setting for fewer than 5 years.

**Results:**

Graduates were generally uncertain about how to develop their career pathways in Hong Kong with few postgraduate development opportunities; because of this some were planning to leave the profession altogether. Despite their expressed needs, they were dissatisfied with the current quality of local PGT and suggested various ways for improvement including supervised practice-based learning, competency-based training, and accreditation of training with trainee involvement in design and evaluation. In addition they identified educational needs beyond TCM, in particular a better understanding of western medicine and team working so that primary care provision might be more integrated in the future.

**Conclusion:**

TCM graduates in Hong Kong feel let down by the lack of public PGT opportunities which is hindering career development. To develop a new generation of TCM practitioners with the capacity to provide quality and comprehensive care, a stronger role for the government, including sufficient public funding, in promoting TCM graduates' careers and training development is suggested. Recent British and Australian experiences in prevocational western medicine training reform may serve as a source of references when relevant program for TCM graduates is planned in the future.

## Background

### i. Development of traditional Chinese medicine in post-colonial Hong Kong

Whilst traditional Chinese Medicine (TCM) has been part of the culture for centuries its official position in both mainland China and Hong Kong has varied. In the mainland it has been officially part of the healthcare system since 1950 when it was promoted by Mao Tse Tung, and remains an integral part of care provision [1,2]. However, in Hong Kong TCM was not recognized as part of the healthcare system until 1997, when the 100 year lease to the UK came to the end and Hong Kong was returned to China[3]. The then newly formed Hong Kong Special Administrative Region (SAR) Government was obliged to enact the requirement laid down in the constitutional law, in which the development of TCM was explicitly stated [4]. A decade has passed since the first Chief Executive of Hong Kong announced the government's commitment to develop TCM in Hong Kong [5] and in his official addresses has reiterated commitment as in the Chief Executive Policy Addresses of 2005 [6], and in the recent 2007 Election Manifesto of the current Chief Executive of the SAR [7]. In the past 10 years, the three major developments in TCM include:

1. Formal regulation and registration of TCM practitioners by the Chinese Medicine Council of Hong Kong (CMCHK), a statutory TCM regulation body established under the Chinese Medicine Ordinance [8].

2. Introduction of TCM services into the public healthcare system, including integrated TCM-western medicine services at both outpatient [9] and inpatient levels[10];

3. Establishment of full time undergraduate training of TCM practitioners in three local universities [11-13], and the implementation of compulsory Continuing Medical Education (CME) program for all registered TCM practitioners, under the auspice of the CMCHK [14].

### ii. Professional TCM education in Hong Kong

New entrants to the TCM profession must obtain formal registration with the CMCHK and this requires the passing of a two part licensing examination with written and clinical components. Currently, only holders of recognized TCM degree(s) are eligible for sitting the examination. Since 1998, undergraduate degree programs have been established in three Schools of Chinese Medicine in public universities. Despite slight differences in structure and emphasis, all three curricula have heavily borrow from TCM universities in mainland China, and include basic western medicine taught along with professional TCM subjects. However, unlike the mainland practice, where graduates are expected to practice both kinds of medicine, Hong Kong TCM graduates, or TCM practitioners from mainland and elsewhere, are not allowed to practice western medicine under the current legislation. For example, TCM practitioners are not permitted to order any diagnostics tests and a western doctor must be involved in any integrated in-patient treatment.

In 2005, a system of continuing education in Chinese medicine (CME) was launched by the CMCHK as an "integral component of the registration system". In each of the three year CME cycles, all registered TCM practitioners must earn 60 CME credits to fulfill their license revalidation requirement. Educational activities are organized by 30 CME program providers accredited by the CMCHK. To be accredited, affiliation with TCM academic departments is not mandatory and many of these providers are private companies [14]. Major components of CME learning include: (a) attending or giving of lectures; (b) submitting reports on independent studies; and (c) publication in TCM journals with or without peer review requirement [15].

In this study, the term *post graduate training *(PGT) refers to both (a) CME and (b) practice based training provided by either public or private sector that are taken by registered Chinese medicine practitioners graduated from the three local Schools of Chinese Medicine.

### iii. Employment and further training for local TCM graduates

In 2003, the first cohort of local university trained TCM practitioners entered the work force but there was a lack of employment opportunities for these graduates. This raised public concern and in the following year, the Legislative councilors twice suggested a stronger role for government in the professional development for TCM graduates [16,17]. Despite prior rhetoric in TCM development, the government's response was that the public sector had no intention of becoming "the sole or major player" in the training of young practitioners, and had assumed the private sector would take a major lead as most of the graduates were expected to "practice in the private sector environment on completion of training" [18]. However, lack of employment opportunities and career pathways continue to be a major issue for TCM graduates [19]. A survey published in 2005 showed that 72.3% of the graduates failed to obtain a full time clinical job after 1 year of graduation [20].

At the time of writing [2008], there are nine TCM clinics within the public sector which are jointly administered by the Hospital Authority (HA), universities, and non-governmental organizations [9]. Despite their institutional identity, these clinics are self-financed and are in line with the government's private sector reliance policy for TCM graduate PGT — only 45 junior posts are made available each year for about 90 new graduates, under a fixed term of 12 months [18,21]. Those who are employed as Junior Chinese Medicine Practitioners receive practice based training in these clinics but there is no published or accredited curriculum to follow. The lack of PGT guidance also applies in private sector training and the quality of training is largely unknown.

### iv. Objective of the study

Despite the government's widely publicized plans for TCM development, its subsequent reluctance to nurture the next generation of TCM practitioners has cast doubts on the quality of PGT, and more importantly, about whether the current system is producing a sustainable TCM workforce that provide quality and safe healthcare for the population. The main objective of this study is to evaluate the perspective of TCM graduates on the effects of current policy by using qualitative methodology. Specifically, we sought graduates' views on their future career prospects and the need for PGT and their opinions on how to improve the current system.

## Method

A purposive sample of 19 TCM graduates with less than 5 years of clinical experience was invited to attend three focus groups held in August and September 2007. Discussants were assembled to ensure the representation of TCM graduates from different universities and with various occupational backgrounds, and thus allowing maximum variation in qualitative data collection [22]. Each discussion consisted of 6–8 participants and lasted for approximately 90 minutes. To enhance the validity and reliability of the analysis, investigators responsible for data analysis acted as the facilitators of all discussions [23]. The discussion followed a semi-structured question guide (Table 1) and was conducted in Cantonese. The sessions were audio taped and transcribed verbatim, and translation was not performed until the writing up of results [24]. Ethics approval was obtained from the university and participants were invited to provide written informed consent prior the discussion. An incentive of HKD$ 200 was paid for the discussant's time and travel.

**Table 1:** Semi-structured question guide used in focus group discussions

1. As a young TCM graduate, how do you look at your career prospect?
2. What are your views on post graduate training in general?

3. What are your opinions on the continuing education in Chinese medicine program?

4. What are your opinions on employer provided training within the public sector?

5. How should post graduate training be modified to suit your needs?

6. Other than TCM, what sort of knowledge should be taught in post graduate training?

The transcripts were analyzed for recurring themes with the aid of NVivo 7 software [25]. A group analytical approach was used to enhance the reliability of the analysis [26]. Specifically, the transcript was read by one investigator (VC) and possible broad themes were identified under the framework of the semi-structured question guide [27]. If these emergent themes occurred repeatedly across and within focus groups, they were noted as recurrent themes. Meanwhile, another investigator (ML) read all the transcripts and generated emergent and recurrent themes independently. Subsequently, the two investigators reached consensus upon emergent themes, then continued to examine the transcripts for connections among these recurrent themes with other investigators via presentation of preliminary results. Groups of related recurrent themes were then organized under a main construct. Interpretations of the themes were illustrated by extracts from the transcripts.

## Results

Analysis of the data generated nine themes and they are clustered under four main constructs, as described in table 2.

**Table 2:** Main constructs and themes identified from the discussions

Main constructs	Themes	Number of codes
A. Current helplessness about local career development	1. Lack of career and professional development opportunities	5
	2. Difficulties in persevering commitment and tendency to leave the local TCM profession	12
B. Views on current post graduate training	1. Need to improve professional competency and status	10
	2. Disappointment with current post graduate training	21
C. Desired reform in post graduate training	1. Supervised, practice based learning style	19
	2. Progressive competency based training linked with career development	13
	3. Training program accreditation and trainees' involvement in design and evaluation	20
D. Expressed educational needs beyond TCM	1. Better knowledge of western medicine for better integration and patient care	32
	2. Better team working with other primary care professionals and better practitioner-patient communication	20

### i. Current helplessness about local career development

#### a. Lack of career and professional development opportunities

As new entrants to the profession, graduates commented that there is a lack of career development opportunities in TCM, private or public sectors alike. They were particularly concerned about the lack of commitment from the government in providing employment opportunities. Graduates admitted that they had low professional esteem due to their lack of competitiveness, especially when compared to the prospects of other western medicine colleagues:

"I always think that we are not valued by the society. Our low salary is a good indicator. My western medicine colleague working in public sector just told me that (s) he is going to have a salary increase of HKD$ 20000. That increment is higher than my current salary...and my pay is lower than a nursing graduate...I am doing this high risk professional job of patient care too...why do I have to accept such a cheap package?" (FG2)

They thought that the lack of sign-posted career pathway had limited their further professional development. In the private sector, more experienced and established TCM practitioners have no incentive in offering mentorship, while public sector training posts are very limited and short in duration:

"If they (the government) are planning to offer post graduate training to us, why there is still no clear direction in doing that?...it's been five years since the first cohort graduated, but there is NO change, NO development, it is getting more and more confused." (FG3)

#### b. Difficulties in persevering commitment and tendency to leave the local TCM profession

Some graduates had aspirations to pioneer local TCM development but meanwhile, hardship and loneliness on this "less traveled" path was anticipated. They were unsure about the professional cohesion amongst the younger generation, and ultimately the possibility of attaining such goals:

"For the future of TCM development, we have to count on ourselves...the very feature of TCM career here is to do it yourself. Improving the overall professional status is the responsibility of the younger graduates, but I am not sure if we are united enough" (FG2)

Other graduates did not share the same enthusiasm and were considering a change in career, if there is no foreseeable improvement in the near future. A few of them had started to pursue formal degrees in other fields, and others expressed interest in practicing overseas:

"I will go anywhere with career opportunities and prospect. It's a pain to gave it (TCM) up but I will only allocate a certain period of time to it...you know youth is limited and I have to consider other aspects like salary, career development and improving my living standards." (FG3)

"I want to be pragmatic with my career and I need to see directions. I will spend 5 more years in TCM and meanwhile will do a part time MBA. If there's an opportunity I will just leave TCM and do business." (FG3)

" I heard that TCM is under professionalization in US and UK...with our training, maybe we can work over there...we are young but local opportunity is very limited...so why can't we have more choices?" (FG2)

### ii. Views on current post graduate training

#### a. Need to improve professional competency and status

Most graduates agreed that PGT is an essential educational process which should facilitate their transition from a supervised intern to an independent practitioner. Such competency escalation should lay a foundation for the acquisition of more advanced skills, and eventually the attainment of specialist qualification. Some graduates pointed out that a formal career management system for graduates is long over due and there is an urgent need to link personal career development and training:

"Career and training should be linked...we should have a better vision for the profession's future...there is no formal training pathway for us to follow now and we will just waste our time learning little in the next decade. Well, I may be named as an "advanced specialist" TCM practitioner in 10 years but so what? If I were teaching by then I will be also wasting the future generation's time. We need this linked pathway immediately. It is kind of late now." (FG3)

It was also recommended that the outcome of PGT should be formally assessed by examinations, which would ensure wider recognition by both the TCM profession and the public. Preferably, completion of basic training should be regarded as the first step in initiating specialist development, and possibly be linked with revalidation of practice license. The pursuit of training and specialist education was seen as a way of "coping with the system", which will potentially place TCM practitioners on a more level playing field with western doctors:

"...Specialist training for TCM...I think this will help TCM practitioners fitting in this western medicine dominated healthcare system...a general TCM internist cannot work in an oncology ward right? You will need some specialist knowledge...western medicine has a specialist examination system but TCM doesn't have it. We need to develop this gradually otherwise we will only worth HK$ 10000 forever." (FG3)

#### b. Disappointment with current post graduate training

##### • Disappointment with CME programs

Almost all discussants did not find the current compulsory CME program useful in improving their capacity in solving day to day clinical problems. Graduates expressed the view that CME system's didactic learning style and repetition of undergraduate materials were more suitable for TCM practitioners without tertiary education:

"(the CME organizer) has organized lectures by some TCM professors from mainland China, but the materials that they covered were just repetition of undergraduate textbooks...I have finished my Bachelor in Chinese Medicine courses and I am very familiar with all this stuff, but this may be useful for those who were not trained in tertiary institutions." (FG1)

They also questioned the effectiveness of CMCHK's quality assurance mechanisms on regulating CME course content, and the control of CME related commercial activities:

"...there is a CME course which you learn how to start up a computer...this is not related to TCM...and has nothing to do with continual medical education! I guess this has to do with the quality monitoring by the government." (FG1)

"...one time the (CME) speaker promoted (his/her) book right after the lecture. Is this acceptable? Would this negatively affect our professional image?" (FG1)

##### • Disappointment with employer provided training within the public sector

Those who received employer provided training raised concerns about the program's supervision style and structure. Trainees said that the quality of TCM apprenticeship varies significantly from mentor to mentor, and questioned whether formal guidance on clinical teaching is in place. When their learning experiences in TCM and western medicine departments were contrasted, the generally positive experience in the latter has made TCM graduates feeling dubious about whether the TCM mentors were adequately incentivised to teach:

"It (the quality of employer provided training) really depends on the training style of each individual TCM mentor, and whether (s) he lets you have hands on experience, and gives you an explanation. I think we have become passive learners under such situations. Sometimes you are learning, but later you may be assigned to do the clerical work of making appointments, or just removing acupuncture needles from the patients. There is no formal structure in supervision, no rules to follow. (FG3)

"Quality of clinical teaching in western medicine is quite impressive. Mentors knew that we need to get trained and were eager to explain in details. But TCM mentorship is the opposite as we are treated like an assistant. They do not recognize their role as a clinical mentor." (FG3)

### iii. Desired reform in post graduate training

#### a. Supervised, practice based learning style

Most graduates would prefer a system of practice based training with feedback on their performance from experienced TCM practitioners. They felt that an active learning style would readily expose their weaknesses and thus allow their accelerated improvement.

"...after prescription you send it over to a senior TCM practitioner, then he or she will amend it for you. This will help us to understand my performance, and gives opportunities for seniors to comment on our work, letting us to get to know our weakness and so we can work on it. We do not want to just sit here and listen." (FG2)

#### b. Progressive competency based training linked with career development

Graduates expected PGT to be an extension of undergraduate learning regardless of its CME or employer provided nature. There was agreement that their needs would best be met by a competency based training syllabus designed to advance trainees' clinical skills progressively in different areas. They expressed their preference for an explicit assessment of the training, with completion of each stage of training linked with staff grade promotion, and eventually attaining a specialist qualification:

"A clear syllabus means...we should have an objective, we need to ensure that we will learn things. In practical terms, this means that there should be structures which guide us in mastering the clinical management of certain conditions." (FG2)

"Will there be a career ladder which we have different (levels) of training each year? Say in the first two years will be a foundation with broad coverage, then in the next two years we can choose to specialize." (FG2)

#### c. Training program accreditation and trainees' involvement in design and evaluation

The option of having a single official accreditation body to monitor the quality of all TCM PGT in the future is supported by most of the gradates. This body would preferably be backed up by academia, and be responsible in granting quotable postgraduate qualification for TCM practitioners in a similar system to that for western medicine training in Hong Kong. Lack of communication channels for voicing views on training issues was also a problem and greater involvement in the design and evaluation of the training program in the future was an option worth exploring.

"The TCM field does not have a Specialist College system (like western medicine does). So we need a similar organization under the CMCHK, or an independent one, to perform central accreditation and quality assurance." (FG-2)

"More senior TCM practitioners maybe willing to voice out our needs but they won't be able to understand us completely...or simply they don't care about our training or career development. We have to get our voice heard. This is a priority." (FG-3)

### iv. Expressed educational needs beyond TCM

#### a. Better knowledge of western medicine for better integration and patient care

When graduates were invited to list areas beyond TCM in which they would find it useful to be trained, most of them named western medicine. They felt that most of their undergraduate western clinical training was in mainland China and they needed to understand the local medical practice better. More importantly, they regarded this as one way to gain recognition in the western dominated Hong Kong healthcare system:

"...during the training we need to transform our understanding of western medicine practice, from the mainland China version to the Hong Kong one. Is this possible? I believe that this will suit our needs...I think this is my major concern." (FG2)

"...Training on western medicine or integrative medicine is just indispensable for modern, young TCM practitioners like us. Western medicine knowledge is very important for us to get accepted in the mainstream healthcare system." (FG3)

The "acceptance" described refers to the facilitation of communication with and referral to western doctors brought about by increased knowledge of western medicine. The desire for more knowledge of western medicine also signifies an attempt to start dialogue with western colleagues, which is expected to foster mutual understanding. The desire to learn western medicine is more than just to gain acceptance. It can also be interpreted as a step in laying the foundations for cooperation and practice of integrative medicine in the future.

"I guess the most important point for learning western medicine is to learn when to refer patients to western doctors. We cannot do everything ourselves." (FG1)

"There was an integrative Chinese – western medicine diabetes workshop which brought both types of practitioners together...it gave a chance for western doctors to understand TCM approaches; and TCM practitioners to ask questions about western treatment...I felt that the atmosphere was very friendly and we were trying to know more about each other, getting a clearer concept on what others were doing." (FG2)

Meeting patients' expectations or demands in day to day clinical practice was another reason cited for the need of further western medicine training. Some graduates mentioned that explaining disease in terms understandable by patients, and informing them on possible treatment options in western medicine should be an integral part of quality TCM training and care. In order to facilitate appropriate referral to or improve cooperation with western doctors, they felt the need to deepen their knowledge in western diagnostic techniques. They expect that further western medicine training would make their ordering of diagnostic tests accepted by medical technologists and other allied healthcare professionals.

"Clinical knowledge in western medicine is very useful. Patients like to bring along their lab reports and radiological films for you to look at, they don't care whether you are Chinese or western medicine practitioner, they just ask for your comments as long as they trust you.. Patients demand, or expect us to give them a reasonable answer. When I have to do this task I feel that my knowledge learnt in the undergraduate years is far from sufficient." (FG 1)

"I always think that there should not be a distinction between Chinese and western medicine. The common subject of interest of all healthcares is patient. I wish my patients will receive the best treatments, but if you are not familiar with western treatment options or you just don't know what the western doctors are doing...then you cannot know how to help the patients. How will you recommend the best treatment options to the patients?" (FG2)

#### b. Better team working with other primary care professionals and better practitioner-patient communication

As general practitioners, graduates expect themselves to be equipped with a broad skill set to provide primary care to patients with different needs. The comprehensive nature of such skill is described as the hallmark of holistic CM practice, and some graduates proposed that substantial experience in general practice should be a prerequisite for specialist training in the future. However, they find it difficult to translate this holistic philosophy into everyday practice, as they find themselves isolated from the rest of the primary care team. Some graduates gave examples of how the current lack of referral mechanisms between TCM practitioners and other healthcare and social service professionals hindered comprehensive patient care. They expected that training in family medicine would empower them to take up a stronger gate-keeping role in primary care:

"Being a TCM practitioner IS being a general practitioner. One may be particularly good at treating some diseases...but still TCM is a kind of holistic medicine." (FG2)

"In principle, TCM practitioners do not look at illness confined in a single part of the body. We should try our best to meet patients' various needs." (FG2)

"Some support from the community is needed. For example if a patient gets services provided by support group then this will help. Or some need to get social security, and some need to get disability allowances. These are real issues but we have no way to find out how we can refer." (FG1)

A particular competency area that raised interest amongst many graduates was doctor-patient communication. It was thought to be an essential skill for gaining trust from the patients, and thus cultivating a continuing, cooperative doctor-patient relationship. They felt that there was a lack of undergraduate education in the training of communication skills and were eager to fill in this knowledge gap:

"One thing I want to learn is how to communicate with patients. There is something special about patients' psychology...how patient response to my question may carry some special meanings. This is not so simple...say he or she may not tell you some of the (medical) histories, but if you have the skills they could have told you so." (FG1)

## Discussion

### i. Principal findings

This study has identified four main career and professional development concerns raised by Hong Kong tertiary trained TCM practitioners who had graduated within the first 5 years of the new training scheme. The lack of sign-posted career pathway has resulted in pessimism amongst these young practitioners and some have thought of leaving the profession. PGT is considered as an integral part of their professional development, but disappointment with both the CME program and employer provided training are expressed. Graduates would prefer an accredited workplace orientated programe which would develop their competency and enable them to escalate their skills and practice. Such a programe needs to be launched in the near future as part of their career development, preferably with their input in both design and evaluation. Knowledge of western medicine and primary care provision were identified as education gaps that hinder their role in providing more comprehensive clinical care, and in gate-keeping of other health related or social services.

### ii. Policy Implications

#### a. Consequences of government' *lassie flair *policy on TCM graduates' professional development

Most of our discussants were uncertain about their future career development and were dissatisfied with their current income. With approximately 90 new graduates looking for clinical positions each year, the size of the problem is increasing. Graduates' tendency to leave clinical practice also implies that there will be a shortage of TCM professionals in the future as new blood fails to replace the aging cohort of TCM practitioners, in which over 35% are 60 years or above [16]. It seems that the investment of public funding in establishing the three Schools of Chinese Medicine is failing to achieve its expected return: a new generation of well trained TCM clinicians who will help achieving the government's goal of integrated TCM development. Our observations suggest that the current graduates lack career pathways, and PGT which could help them develop their competencies should not be confined in TCM but also western medicine. Medical education has long been recognized to be a continuum [28] and thus current policy on the graduates' professional development needs to be reviewed to make it fit for purpose.

#### b.TCM private sector as the major provider of post graduate training: Is it appropriate?

Relying on the private sector to provide junior clinical posts and high quality training for fresh TCM clinicians is unrealistic in Hong Kong for both infrastructural and academic reasons. First, most TCM practitioners in the private sector work in solo practices in the community [29] and thus have no capacity or economic incentives for teaching junior TCM practitioners. The second reason is the differences in academic backgrounds between private sector practitioners and graduates, in which the majority of the former acquired their skills through unstructured apprenticeship of varying standards[29] which poses a question on the academic and teaching capacity of the private sector in providing postgraduate teaching. This notion is supported by graduates' negative comments towards CME programs, which are mainly provided by small TCM organizations in the private sector [30]. We recommend the CMCHK should strengthen their quality assurance efforts and improve the provision of CME programs that can better cater to the needs of graduates. In addition, evidence of the questionable effectiveness of lecture based CME programs has long been established [31]. The CMCHK should initiate a reform of the CME system to accommodate a more effective Continual Professional Development (CPD) in the pedagogical mode in the future, which would be more compatible with the graduates' needs and learning style [32].

#### c. Current financial constraints in the public sector's role

In view of the worsening situation, the government has shown some early signs in changing its policy direction. The Secretary for Food and Health has announced the opening of more TCM outpatient clinics within the public sector to cater for the needs of graduate training [33]. But before these plans are implemented, current public sector training will require a considerable make over to achieve the goals of proper career management. Current arrangements only allow half of all graduates to be employed on a one year contract. Under this financing context, establishing a sign-posted career pathway linking training and personal advancement is almost impossible and the HA has openly admitted that these clinics' hiring capacity is limited [21]. International experience has shown that the impact of public funding on graduate medical training is substantial [34]. Securing a dedicated funding for TCM graduates professional development would be the foremost issue to be resolved.

#### d. Provision of multi-disciplinary education for TCM graduates: ethical obligations

The need for inter-professional education for newly graduated TCM clinicians provides the rationale for public sector intervention as it directly influences quality of care. Our results illustrated that graduates regard the acquisition of western medicine knowledge as a catalyst for the development of integrative medicine, and more importantly, it relates to the ethical issue of empowering patient in making informed choice between western and Chinese treatment options. The views of graduates are vividly triangulated when the public's utilization pattern is taken into account, since TCM is often used in conjunction with western medicine, especially amongst chronic disease patients [35]. The existing western medicine education system within the public sector would be an ideal venue for east-west interdisciplinary learning, and its potential benefit could also be extended to western medical students who have little knowledge and exposure to TCM practice [36]. In a society in which the public choose both modalities of care it makes sense to promote greater understanding amongst the totality of the physician community.

Another instrumental role that the public sector should play is to ally TCM graduates with the wider primary care team via multi-professional education. Currently, 15% of all outpatient services are provided by TCM practitioners and their clients are often older chronic disease patients with relatively lower quality of life[37]. It is evident from our findings that the graduates are experiencing difficulties in providing referral services for patients with needs beyond TCM care, and thus preventing comprehensive primary care provision. In line with our recommendations, one of the major themes in the government's recent proposal in healthcare delivery reform is to foster collaboration between healthcare and social services professionals in the community. Equipping TCM gradates with appropriate knowledge of western medicine and primary care provision is an ethical obligation that the government and health authorities should not overlook. Last but not least, the complicated health needs profile of TCM services users also warrant the enhancement of clinical communication skills training amongst TCM graduates, as patient-practitioner relationship is found to be a critical determinant in the perceived outcomes of CAM treatment [38].

#### e. Career management for TCM graduates: Lessons learned from western medicine

If the government were to develop career and training management for TCM graduates in the future, the recent developments in prevocational western medicine training in the UK would be an invaluable source of reference [39]. The directions for reform mentioned by our interviewees coincide with some of the core elements in this newly adopted training approach in western medicine. For example, the 2 year UK Foundation Year Program (FYP) for fresh medical school graduates has highlighted the pivotal role of supervision in practice based learning, and accordingly, protected time and resources are allocated to ensure quality of teaching by senior doctors. Guided by a trainee centred orientation, learning objectives are clearly laid out to facilitate the attainment of clinical competency. Learning outcomes are evaluated with a series of structured assessments, and satisfactory completion of the FYP is considered as a prerequisite for the entrance of specialist training program. Finally, the task of accreditation and implementation of the FYP is officially designated to the Postgraduate Medical Education Training Board and Postgraduate Deaneries at each NHS Trusts respectively. Overall, the strength of the new UK system lies in its clarity in curricular, infrastructure and implementation method. Similar frameworks have been adopted in the Australian Curriculum Framework for Junior Doctors [40]. Of course, the fundamental differences between western medicine and TCM would clearly inhibit a direct transplantation of the FYP into a TCM setting. Nevertheless, the FYP's overall approach and infrastructure may prove worthwhile in the design of professional development program for TCM graduates. More importantly it could be adopted for integrated training for both TCM and western trained junior doctors.

#### f. Recommendations

In summary, whilst the policy direction towards a more integrated and highly developed TCM profession has been clearly laid out, the process to achieve the integration, particularly formal post graduate medical education, has yet to be put in place. The views of the TCM graduates provide a clear steer for their requirements. To maximize the resources available to the population in Hong Kong these views should be taken into account by those who are able to make the necessary changes. In addition, although we have focused on Hong Kong, there will be lessons for policy makers in other countries. While regulation is the first step in ensuring the quality and safety of CAM practices, experience from Hong Kong shows that policy and public investment for fostering continual professional development is necessary for building a sustainable CAM workforce. Involvement of the western medicine sector in the training of CAM professionals could enhance multidisciplinary learning and subsequently promoting teamwork between CAM and western medicine professionals. Finally, the design of such development program could benefit from emulating the structured approach in the training of junior western doctor. Further case study researches in other health systems are warranted to test the generalizability of our findings.

## Completing interests

The authors declare that they have no competing interests.

## Authors' contributions

VC, ML and SG conceived the research idea. VC and ML conducted the focus group interviews. All authors involved in data analysis. SW and SM added critical comments on the interpretations of data and on the manuscript. SG supervised the whole research process.

## Pre-publication history

The pre-publication history for this paper can be accessed here:


